# Atorvastatin prevents age-related and amyloid-β-induced microglial activation by blocking interferon-γ release from natural killer cells in the brain

**DOI:** 10.1186/1742-2094-8-27

**Published:** 2011-03-31

**Authors:** Anthony Lyons, Kevin J Murphy, Rachael Clarke, Marina A Lynch

**Affiliations:** 1 Trinity College Institute for Neuroscience, Physiology Department, Trinity College, Dublin 2, Ireland

## Abstract

**Background:**

Microglial function is modulated by several factors reflecting the numerous receptors expressed on the cell surface, however endogenous factors which contribute to the age-related increase in microglial activation remain largely unknown. One possible factor which may contribute is interferon-γ (IFNγ). IFNγ has been shown to increase in the aged brain and potently activates microglia, although its endogenous cell source in the brain remains unidentified.

**Methods:**

Male Wistar rats were used to assess the effect of age and amyloid-β (Aβ) on NK cell infiltration into the brain. The effect of the anti-inflammatory compound, atorvastatin was also assessed under these conditions. We measured cytokine and chemokine (IFNγ, IL-2, monocyte chemoattractant protein-1 (MCP-1) and IFNγ-induced protein 10 kDa (IP-10)), expression in the brain by appropriate methods. We also looked at NK cell markers, CD161, NKp30 and NKp46 using flow cytometry and western blot.

**Results:**

Natural killer (NK) cells are a major source of IFNγ in the periphery and here we report the presence of CD161^+ ^NKp30^+ ^cells and expression of CD161 and NKp46 in the brain of aged and Aβ-treated rats. Furthermore, we demonstrate that isolated CD161^+ ^cells respond to interleukin-2 (IL-2) by releasing IFNγ. Atorvastatin, the HMG-CoA reductase inhibitor, attenuates the increase in CD161 and NKp46 observed in hippocampus of aged and Aβ-treated rats. This was paralleled by a decrease in IFNγ, markers of microglial activation and the chemokines, MCP-1 and IP-10 which are chemotactic for NK cells.

**Conclusions:**

We propose that NK cells contribute to the age-related and Aβ-induced neuroinflammatory changes and demonstrate that these changes can be modulated by atorvastatin treatment.

## Background

Microglia express a vast array of receptors [1] indicative of the many factors which can increase their activation. One of the most potent activators of microglia is IFNγ and its concentration has been shown to increase in the brain of aged, Aβ-treated and LPS-treated animals [2,3]. IFNγ may act as an endogenous activator of microglial activation but whether it can be released from resident cells in the brain is not clear, although it has been reported that it is produced by microglia of *Toxoplasma gondii*-infected severe combined immunodeficiency (scid) mice [4].

Natural killer (NK) cells are involved in immune surveillance and responsible for the initial response to viral and pathogenic infection. They express receptors for several cytokines including IL-2 which triggers cell proliferation, cytotoxic activity and release of cytokines, including IFNγ [5].

Statins, inhibitors of HMG-CoA reductase, suppress IL-2-induced cytotoxicity of NK cells [6] and attenuate the age-related and Aβ-induced microglial activation, and accompanying increase in IFNγ [3,7]. Here we investigated the possibility that IFNγ released by infiltrating CD161^+ ^cells might contribute to microglial activation in 2 neuroinflammatory models, the aged rat and the Aβ-treated rat. We also investigated whether the modulating effect of atorvastatin may be a consequence of its ability to interfere with release of IFNγ from these infiltrating cells. The evidence presented here suggests that IFNγ plays a role in the age-related and Aβ-induced increase in microglial activation and that the source of the IFNγ is CD161^+ ^cells. Importantly, treatment with atorvastatin decreases CD161 and NKp46 (a specific NK cell marker) in the hippocampus of aged and Aβ-treated animals and consequently decreases the changes in IFNγ and microglial activation.

## Methods

### Animals and study design

Male Wistar rats (Bantham and Kingman, UK; n = 5-7) aged 3 months (250-350 g) or 22 months (550-650 g), housed in pairs or groups of 4 respectively. All these experiments were performed under license (Department of Health, Ireland) and in accordance with local ethical guidelines.

In the first study, young and aged rats were anaesthetized with urethane (1.5 g/kg intraperitoneally) and transcardially perfused with 200-300 ml saline before tissue was harvested. In the second study, young and aged rats (n = 5-7) were divided into a control group and a group that received atorvastatin orally (5 mg/kg per day) for 8 weeks. Mean body weights of young animals (g) before and after treatment were 222 ± 9 and 356 ± 15 in the control group and 219 ± 7 and 340 ± 13 in the atorvastatin-treated group; the corresponding weights in aged rats were 534 ± 17 and 555 ± 22 (controls) and 527 ± 16 and 577 ± 22 (atorvastatin). In the third study, young rats, assigned to a control- or an Aβ-treated group, were subdivided into rats which did/did not receive atorvastatin (5 mg/kg per day) for 3 weeks; mean body weights (g) before and after treatment were 291 ± 12 and 337 ± 9 in the control group and 277 ± 13 and 336 ± 15 in the atorvastatin-treated group.

Ground atorvastatin calcium tablets (Lipitor™; Pfizer-Parke Davis, Ireland) were dissolved in vegetable oil (5 ml/150 g chow) and added to laboratory chow (Red Mills, Ireland); control animals received laboratory chow with added vegetable oil. The dose of atorvastatin was chosen on the basis of previous findings [3,8]. Food intake (18 g and 25 g per rats per day for young and aged rats respectively) was assessed before and during the study to ensure animals received their full atorvastatin dose and fresh food was prepared every 2 days. Mean atorvastatin concentrations (± SEM; ng ATV/mg protein) in plasma and liver respectively were 0.008 ± 0.002 in young rats and 0.005 ± 0.002 in aged rats, and 0.026 ± 0.05 in young rats and 0.020 ± 0.05 in aged rats.

Aβ_1-42 _(5 μl; 200 μM; aggregated for 48 hours at 37°C; Biosource International Inc. US) or vehicle was delivered intracerebroventricularly (2.5 mm posterior and 0.5 mm lateral to bregma; depth 3.5 mm) to urethane-anaesthetized rats [7], on the last day of atorvastatin treatment. [We have previously established that treatment of animals with reverse peptide, Aβ_40-1 _exerted no effect on hippocampal expression of MHCII, CD40 or CD11b mRNA, or on hippocampal IL-1β at mRNA or protein level]. Rats were killed 4 hours after Aβ injection [7], the hippocampus was dissected and used for preparation of dissociated cells by flow cytometry or for analysis of mRNA or for western immunoblot.

### Assessment of NK cells by flow cytometry

Following transcardial perfusion hippocampal tissue was harvested and passed through a cell strainer (70 μm) and centrifuged (170 × g, 10 min). The pellet was resuspended in PBS containing collagenase D (1 mg/ml) and DNase I (200 μg/ml), incubated at 37°C for 30 min and centrifuged (170 × g, 5 min). Pellets were resuspended in 1.088 g/ml Percoll (9 ml), underlaid with 1.122 g/ml Percoll (5 ml), and overlaid with 1.072 g/ml and 1.030 g/ml (9 ml each) Percoll and PBS (9 ml) and centrifuged (1250 × g, 45 min). The mononuclear cells (between the 1.088:1.072 and 1.072:1.030) were centrifuged and the pellets were washed, and blocked for 15 min at room temperature in FACS block (Mouse BD Fc Block (BD Pharmingen, UK); 1:500 in FACS buffer). Cells were incubated with CD161a-FITC (1:100 in FACS buffer; BD Biosciences, UK) and NKp30-PE (1:30 in FACS buffer; Santa Cruz Biotechnology Inc, USA). Immunofluorescence analysis was performed on a DAKO Cyan ADP 7 colour flow cytometer (DAKO Cytomation, UK) with Summit v4.3 software.

### Isolation of NK cells

IgG-precoated magnetic beads (100 μl; 4 × 10^6 ^beads; monoclonal human anti-mouse IgG; Invitrogen, UK) were incubated overnight at 4°C with mouse anti-rat CD161 primary antibody (1 mg/ml: Serotec, UK) or mouse IgG (0.4 mg/ml; Santa Cruz, USA). Perfused hippocampal or cortical tissue was triturated in pre-warmed DMEM (Gibco BRL, Scotland), centrifuged (2000 × g, 4 min) and trypsinized (Tryple Express; Invitrogen, UK; 2 min, RT). Cells were centrifuged (2000 × g, 4 min), resuspended in DMEM and filtered 3 times, incubated with CD161-coated or IgG-coated beads (2 hours, 4°C) and collected by magnetic isolation. Cell isolation was checked by gel electrophoresis, and visualized following western immunoblot using a monoclonal anti-CD161 antibody (Serotec, UK) and a HRP-linked secondary antibody. These CD161^+ ^cells were incubated with/without IL-2 (100 ng/ml; Biosource, USA) for 24 hours and IFNγ was assessed in supernatants.

In another experiment, a human NK cell line was grown in RPMI with glutamax (Gibco, UK) supplemented with foetal calf serum (10%; Gibco, UK), penicillin (100 U/ml; Gibco, UK), streptomycin (100 μg/mg; Gibco, UK) and IL-2 (20 ng/ml; R&D Systems, UK). Cells were washed (1200 g × 10 min) 3 times, resuspended in warmed media and incubated for 24 hours in the presence/absence of IL-2 (20 ng/ml). Supernatants were assessed for IFNγ by ELISA.

### Preparation of mixed glial cultures

Primary cortical mixed glia were prepared from 1 day old male Wistar rats as previously described [2]. Briefly cortical tissue was chopped, incubated for 5 minutes in 5% CO_2 _at 37°C in warm filter-sterilized Dulbecco's Modified Eagle Medium (DMEM; Sigma Aldrich, UK) supplemented with penicillin (100 μl/ml; Gibco, UK), streptomycin (100 μl/ml; Gibco, UK) and FBS (10% w/v; Gibco, UK). A single cell suspension was prepared by triturating tissue, filtering though a 40 μm sterile nylon mesh filter (BD Biosciences, USA), centrifuging at 2000 *g *for 3 minutes at 20°C, and resuspending the pellet 1 ml warm filter-sterilized culture media. Tissue was triturated and resuspended glia were counted, equal numbers of cells were plated onto 6 well plates and incubated for 2 hours in 5% CO_2 _at 37°C to allow the cells to adhere. After 2 hours the cells were flooded with 1 ml warm culture media and incubated for 3 days in 5% CO_2 _at 37°C. Culture media was replaced with fresh culture media every 3 days for 12 days until cells were ready for treatments. After 12 days in culture, cells were incubated in the presence or absence of IFNγ (50 ng/ml; 24 hours at 37°C; R&D Systems, UK). This concentration has been shown to robustly increase MHCII mRNA [9]. Cells were harvested for analysis of MHCII and CD11b mRNA.

### Real-time PCR

Total RNA was extracted from snap-frozen hippocampal tissue or cultured glial cells using a NucleoSpin^® ^RNAII isolation kit (Macherey-Nagel Inc., Germany). Total RNA concentrations were determined by spectrophotometry, samples were equalised and cDNA synthesis was performed on 1 μg total RNA using a High Capacity cDNA RT kit (Applied Biosystems, Germany). Real-time PCR was performed using Taqman Gene Expression Assays (Applied Biosystems, Germany); the assay IDs for MHCII, CD11b, MCP-1 and IP-10 were Rn01768597_m1, Rn00709342_m1, Rn00580555_m1 and Rn00594648_m1, respectively. Real-time PCR was conducted using an ABI Prism 7300 instrument (Applied Biosystems, Germany). A 20 μl volume was added to each well (9 μl of diluted cDNA, 1 μl of primer and 10 μl of Taqman^® ^Universal PCR Master Mix). Samples were assayed in duplicate (40 cycles; 95°C for 10 min, 95°C for 15 sec for each cycle (denaturation) and the transcription step at 60°C for 1 min). β-actin was the endogenous control (VIC-labeled MGB Taqman probe, Applied Biosystems, Germany; Assay ID: 4352341E). Gene expression was calculated relative to the endogenous control samples and to the control sample giving an RQ value (2^- ^^DDCt^, where CT is the threshold cycle)

### Analysis of CD161 and NKp46 by western blot

Hippocampal tissue was equalized for protein, aliquots (10 μl) were loaded onto 10% gels, separated (30 mA; 60 min) and transferred onto nitrocellulose (225 mA; 90 min). Proteins were immunoblotted over night hours at 4°C with anti-CD161 antibody (1:500 in TBS containing 0.05% Tween-20/2% BSA; Serotec, UK) or anti-NKp46 antibody (1:500 in TBS containing 0.05% Tween-20/2% BSA; R&D Systems, UK). Membranes were washed, incubated with the HRP-linked secondary antibody (1:1000; anti-mouse IgG; Sigma, UK) Immunoreactive bands were detected with ECL (GE Healthcare, UK) chemiluminescence. Membranes were then stripped with Re-blot (Re-blot Plus Strong Solution; Chemicon, UK) for 10 min at room temperature (1:10 dilution; Chemicon International, US) and probed for actin expression (Sigma, UK). Bands were quantified relative to the endogenous control using densitometry (Labworks, UVP BioImaging Systems, UK).

### Analysis of IFNγ and IL-2

Cytokines were assessed by ELISA in hippocampal tissue prepared from rats and, in the case of IFNγ, also in a human NK cell line. For IFNγ, 96-well plates were coated with monoclonal mouse anti-rat or anti-human antibody. Pre-coated 96-well plates (BioSource, US) were used for analysis of IL-2. Samples or standards, and subsequently detection antibody (biotinylated goat anti-rat or anti-human antibody), were added and after incubation in the presence of streptavidin-horseredish peroxidase, substrate solution was added [2]. Cytokine concentrations were evaluated with reference to standard curves.

### Statistical analysis

Data were analyzed using either Student's t-test for independent means, or a 1 way ANOVA followed by post hoc Student Newman-Keuls test. Data are expressed as means of between 5 and 7 replicates (± SEM).

## Results

### Age increases microglial activation and IFNγ production in the brain

**Figure 1:**
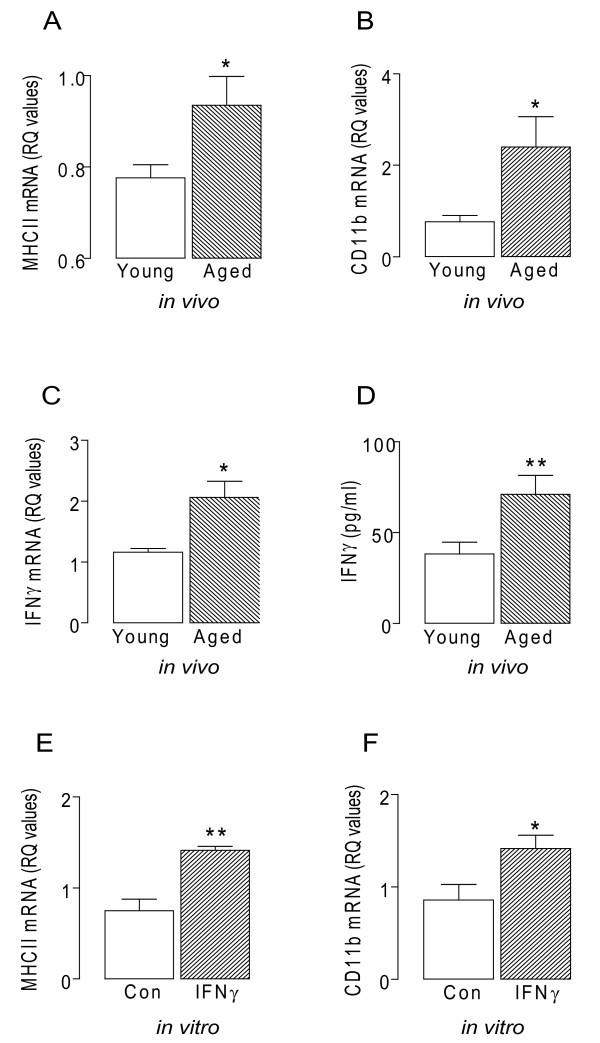
**Age related inflammatory changes in the brain**. A, MHCII mRNA B, CD11b mRNA C, IFNγ mRNA expression, and D, IFNγ concentration were significantly increased in hippocampal tissue prepared from aged compared with young rats (*p < 0.05; **p < 0.01; student's t-test for independent means; n = 5-7). E, IFNγ (50 ng/ml) significantly increased MHCII mRNA and F, CD11b mRNA in cultured glia *in vitro*.

Here we assessed expression of cell surface markers of microglial activation, and demonstrate age-related increases in MHCII and CD11b mRNA in hippocampal tissue, consolidating earlier findings [10,11]. These changes are accompanied by significant increases in IFNγ mRNA and protein (p < 0.05 or p < 0.01; student's t-test for independent means; Figure 1A-D). IFNγ is a potent activator of microglia [12,13] and, consistent with this, we show that incubation of mixed glia in the presence of IFNγ significantly increased MHCII and CD11b mRNA (p < 0.05 or p < 0.01; student's t-test; Figure 1E, F).

### Age increases IFNγ producing-CD161^+ ^NK cells in the brain

**Figure 2:**
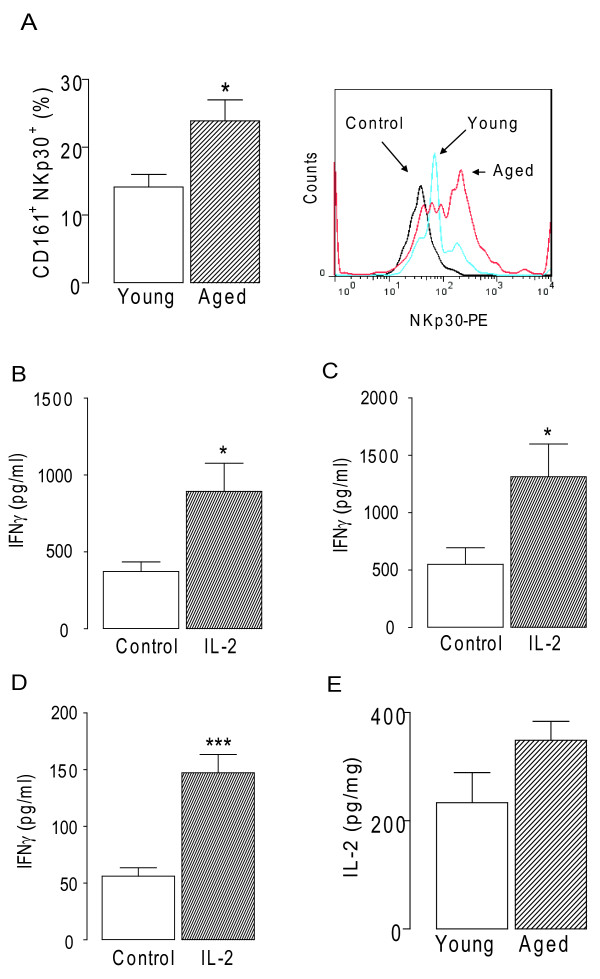
**Age-related NK cell infiltration into the brain**. A, CD161^+ ^NKp30^+ ^cells was increased in the hippocampus of aged compared with young rats (*p < 0.05; student's t-test for independent means; n = 6). The panel to the right in Figure 2b shows a sample flow cytometry plot, demonstrating increased expression of NKp30 in the aged brain, after gating on the CD161^+ ^cells. B, IL-2 increased IFNγ release from CD161^+ ^cells obtained from rat hippocampus and C, cortex, and D, from a human NK cell line (*p < 0.05; ***p < 0.001; student's t-test for independent means; n = 6). E, IL-2 concentration was not significantly altered in hippocampus of aged and young rats.

The resident cell source of IFNγ in brain remains unclear. In numerous experiments, we have been unable to stimulate its release with IL-12, IL-2 and IL-18, or LPS from cultured mixed glia or neurons; however, one possible cell source of IFNγ is NK cells. The data presented here indicate that there was an age-related increase in the number of CD161^+ ^NKp30^+ ^cells in the brain (p < 0.05; student's t-test; Figure 2A), thus suggesting the specific presence of NK cells. The panel to the right in Figure 2A shows a sample flow cytometry plot, demonstrating increased expression of NKp30 in the aged brain, after gating on the CD161^+ ^cells.

We prepared CD161^+ ^cells from hippocampal and cortical tissue, stimulated them with IL-2 and show that IFNγ release was increased between 2 and 3 fold in each case (p < 0.05; student's t-test for independent means; Figures 2B, C) which compares with significant IL-2-induced release from a human NK cell line (p < 0.001; student's t-test; Figure 2D). However no statistically significant age-related change in IL-2 was observed (Figure 2E).

### Atorvastatin attenuates age-related and Aβ-induced CD161 expression

**Figure 3:**
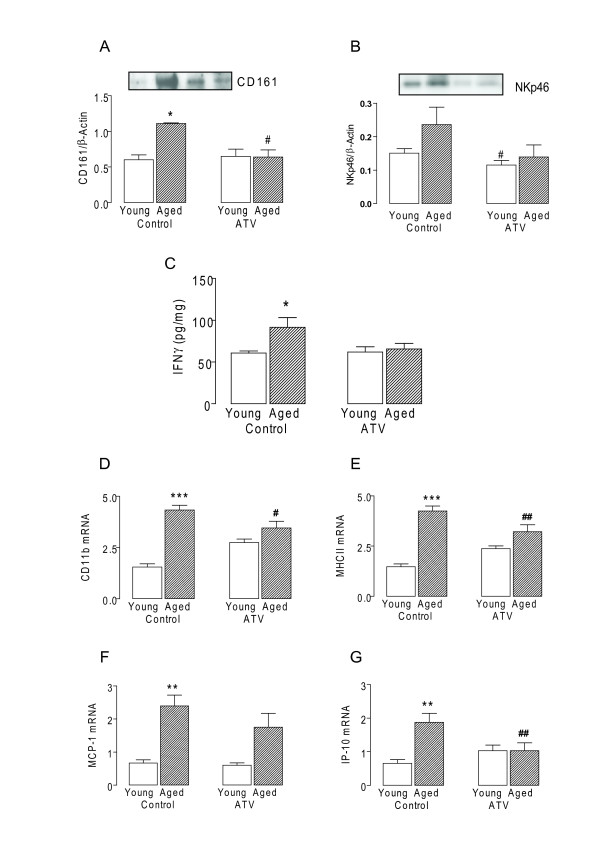
**Age-related NK cell induced changes are attenuated by Atorvastatin**. A, Expression of CD161 and B, NKp46 assessed by western immunoblotting. Densititometry data is expressed in arbitrary units relative to β-Actin C, IFNγ expression, D, CD11b mRNA, E, MHCII mRNA, F, MCP-1 mRNA and G, IP-10 mRNA (*p < 0.05; **p < 0.01; ***p < 0.001; ANOVA; n = 6-7) were significantly increased in hippocampal tissue prepared from aged, compared with young rats. These changes were attenuated in hippocampal tissue prepared from aged rats which received atorvastatin (ATV; 5 mg/kg per day) for 8 weeks (ATV; ^#^p < 0.05; ^##^p < 0.01; vs aged rats which did not receive atorvastatin; ANOVA).

The data indicate that there were age-related increases in expression of CD161 (p < 0.05; ANOVA; Figure 3A) and another marker of NK cells, NKp46 (Figure 3B). This was coupled with increased IFNγ (p < 0.05; ANOVA; Figure 3C) in the aged brain and these changes were attenuated in tissue prepared from aged rats treated with atorvastatin. Similarly the significant age-related increases in CD11b and MHCII, and the chemokines, MCP-1 and IP-10 (p < 0.01 or p < 0.001; ANOVA; Figure 3D-G), which are chemotactic for NK cells [14], were also attenuated in hippocampal tissue prepared from atorvastatin-treated aged rats (p < 0.05 or p < 0.01; ANOVA).

**Figure 4:**
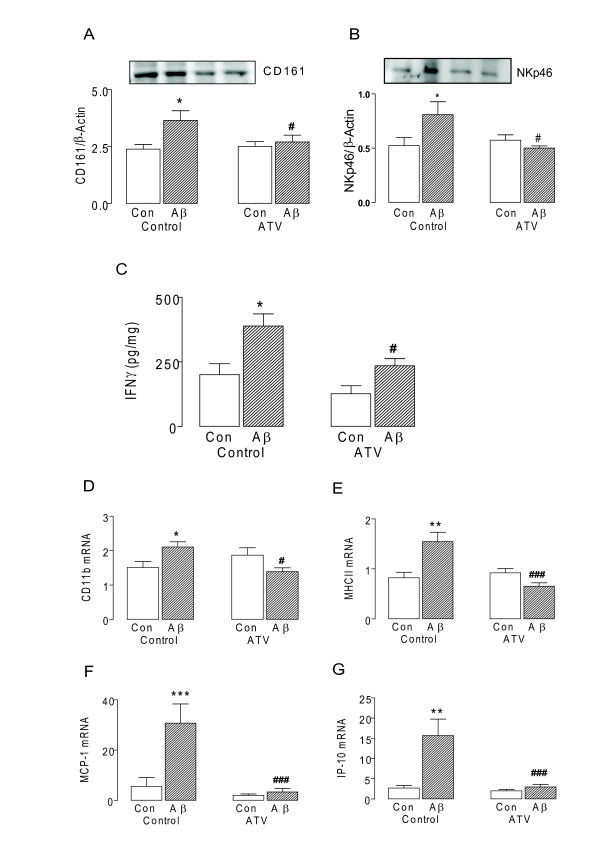
**Aβ-related NK cell induced changes are attenuated by Atorvastatin**. A, Expression of CD161 and B, NKp46 assessed by western immunoblotting. Densititometry data is expressed in arbitrary units relative to β-Actin C, IFNγ concentration, D, CD11b mRNA, E, MHCII mRNA, F, MCP-1 mRNA and G, IP-10 mRNA (*p < 0.05; **p < 0.01; ***p < 0.001; ANOVA; n = 6-7) were significantly increased in hippocampal tissue prepared from rats which received an intracerebroventricular injection of Aβ_1-42 _(5 μl; 200 μM) or vehicle. These changes were attenuated in hippocampal tissue prepared Aβ-treated rats which received atorvastatin (ATV; 5 mg/kg per day) for 3 weeks (ATV; ^#^p < 0.05; ^###^p < 0.001; vs Aβ-treated rats which did not receive atorvastatin; ANOVA).

In parallel, we observed that acute inflammation induced by intracerebroventricular injection of Aβ, caused a significant increase in expression of CD161 and NKp46 (p < 0.05; ANOVA; Figure 4A, B), This was again correlated with increased IFNγ expression in the aged brain (p < 0.05; ANOVA; Figure 4C). These changes were attenuated in tissue prepared from aged rats treated with atorvastatin (p < 0.05; ANOVA; Figure 4A-C). Aβ-induced increases in CD11b and MHCII, and the chemokines, MCP-1 and IP-10 (p < 0.01 or p < 0.001; ANOVA; Figure 4D-G), were also attenuated in rats treated with atrovastatin (p < 0.05 or p < 0.001; ANOVA).

## Discussion

The significant and novel findings of this study are that CD161-positive cells are observed in the brain under inflammatory conditions, that these cells release IFNγ. This results in microglial activation, and that treatment of aged or Aβ-injected rats with atorvastatin reduces CD161 and NKp46 expression and the consequent increases in IFNγ and microglial activation.

Age-related increases in MHCII and CD11b presented here mirror earlier findings [10], as does the parallel increase in IFNγ [12]. It is well known that IFNγ increases microglial activation and its effect on MHCII and CD11b is similar to that described elsewhere [12], however, key questions remain as to whether resident cells in the brain are capable of producing IFNγ and whether endogenous IFNγ stimulates microglial activation. It appears that resident brain cells produce IFNγ only in very specific circumstances. IFNγ transcripts have been identified in human tumour neuroglia and human astroglial cell lines [15] and in microglia prepared from athymic nude and scid mice infected with Toxoplasma gondii [4]. However, we have been unable to detect release of IFNγ from cultured glia or neurons following stimulation with LPS or with IL-2, IL-12 or IL-18 alone or in combination with LPS. Therefore, we have considered that NK cells, which are known to be a major source of IFNγ, might be present in brain and that these cells may be a source of IFNγ. The data presented here show the presence of NK cell markers, CD161 and NKp46 in the aged hippocampus. The presence of NK cells was then further suggested by flow cytomerty, showing increased numbers of CD161^+^NKp30^+ ^cells in the aged hippocampus.

CD161 is one of the archetypal markers of NK cells, and NKp30 and NKp46 are activating receptors present on the surface of NK cells [16]. However, it is acknowledged that CD161 is also on a subset of CD4^+ ^and CD8^+ ^T cells [17] but previous work has shown that T cells are only present in very low numbers in brain [18]. These data presented here suggest that infiltration of cells occurs more readily in the brain of aged and Aβ-treated rats compared with young rats. This may be a consequence of the reported inflammatory-mediated increase in blood brain barrier (BBB) permeability [19], an increased change in molecules chemotactic for NK cells or both. Interestingly the age-related and Aβ-induced increase in CD161 and NKp46 is paralleled by increased hippocampal expression of IP-10 and MCP-1, both of which have been shown to promote chemotaxis of NK cells [14,20].

IL-2, IL-12 and IL-18 all potently stimulate release of IFNγ from NK cells [21] and here we show that IL-2 increases IFNγ release from CD161^+ ^cells prepared from cortex and hippocampus. However, IL-2 expression in hippocampus was not altered significantly with age suggesting that the increase in IFNγ results from the increased infiltration of NK cells into the brain rather than any change caused by IL-2.

Atorvastatin decreased CD161 and NKp46 expression in the brain of aged rats, suggesting that NK cell numbers in the brain may be decreased. Interestingly atorvastatin has been shown to increase patency of the BBB in a hypertension model, by increasing expression of two tight junction proteins, zonula occludens-1 and occludin [22]. The present findings, which indicate that atorvastatin decreased the age-related expression of MCP-1 and IP-10, suggest that the atorvastatin-induced loss of the chemotactic signal may also contribute to the reduction in CD161 and NKp46. Consistent with our proposal that IFNγ, derived from NK cells, contributes to microglial activation in the brain of aged rats, we show that the atorvastatin-associated change in CD161 and NKp46 was accompanied by an attenuation of the age-related changes in MHCII, CD11b and IFNγ; these changes precisely mirror our earlier observations [3]. It is interesting to note that atorvastatin treatment alone caused a slight increase in expression of CD161, CD11b, MHCII, and IP-10, although this increase did not reach statistical significance; these effects are apparently independent of IFNγ and NKp46. One possibility may be that atorvastatin decreased coenzyme Q10 expression which is known to increase inflammation [23]. The fact that these chages were evidence only when rats were treated with atorvastatin for 8 weeks, rather than 3 weeks, reflects the finding that changes in coenzyme Q10 appear to be dependent on the type of statin used and the duration of the treatment {Marcoff, 2007 #1940}.

The age-related increase in CD161 and NKp46 was replicated by intracerebroventricular injection of Aβ, suggesting that NK cells also infiltrate the brain in this model. BBB permeability has been shown to be compromised in a mouse model of Alzheimer's disease [24], while soluble Aβ has been shown to stimulate inflammatory changes in the vasculature [25] and to permit monocyte infiltration into the brain [26]. The Aβ-induced increase in CD161 and NKp46 was paralleled by microglial activation and chemokine production, mirroring the age-related changes. Atorvastatin attenuated the Aβ-induced changes as it did the age-related changes, and this replicates some of our earlier findings [7]. The modulatory effects of atorvastatin on microglial activation may provide some insight into why epidemiological evidence suggests that statins reduce the incidence of AD; one possibility is that its primary effect is on correcting any compromise in BBB permeability [22] and consequently prevents infiltration of NK cells, that lead to subsequent microglial activation and inflammatory changes.

## Conclusion

The findings described here provide the first evidence that cells which have a number of phenotypic characteristics of NK cells are present in the aged brain, and the data suggest that these cells may release IFNγ which acts to stimulate microglial activation. This microglial activation can be reversed by treatment with atorvastatin which works by down-regulating the expression of IP-10 and MCP-1, chemoattractant molecules for NK cells.

## Competing interests

The authors declare that they have no competing interests.

## Authors' contributions

AL and KJM contributed to design of the study, performed the cell isolation western blot, Real-Time PCR and FACS analysis, and reviewed and organized the data; RC performed all atrovastatin related animal treatments, tissue preparation, IFNγ ELISAs and reviewed the manuscript; MAL directed the overall study, analysis of the data, wrote and reviewed the manuscript. All authors have read and approved the final manuscript.
